# Diverse in Local, Overlapping in Official Medical Botany: Critical Analysis of Medicinal Plant Records from the Historic Regions of Livonia and Courland in Northeast Europe, 1829–1895

**DOI:** 10.3390/plants11081065

**Published:** 2022-04-13

**Authors:** Julia Prakofjewa, Martin Anegg, Raivo Kalle, Andra Simanova, Baiba Prūse, Andrea Pieroni, Renata Sõukand

**Affiliations:** 1 Department of Environmental Sciences, Informatics and Statistics, Ca’ Foscari University of Venice, Via Torino 155, Mestre, 30172 Venezia, Italy; yuliya.prakofyeva@unive.it (J.P.); martin.anegg@gmx.at (M.A.); baiba.pruse@unive.it (B.P.); renata.soukand@unive.it (R.S.); 2 University of Gastronomic Sciences, Piazza Vittorio Emanuele II 9, 12042 Pollenzo, Italy; a.pieroni@unisg.it; 3 Department of Latvian and Baltic Studies, University of Latvia, Visvalža Str. 4a, LV-1050 Riga, Latvia; andra.simanova@inbox.lv; 4 Institute for Environmental Solutions, “Lidlauks”, Priekuļi Parish, LV-4126 Cēsis, Latvia; 5 Medical Analysis Department, Tishk International University, 100 Meter Street & Mosul Road, Erbil 44001, Iraq

**Keywords:** historical ethnobotany, local ecological knowledge, old herbals, scholarly medicine, Livonia, Courland

## Abstract

Works on historical ethnobotany can help shed light on past plant uses and humankind’s relationships with the environment. We analyzed medicinal plant uses from the historical regions of Livonia and Courland in Northeast Europe based on three studies published within the 19th century by medical doctors researching local ethnomedicine. The sources were manually searched, and information extracted and entered into a database. In total, there were 603 detailed reports of medicinal plant use, which refer to 219 taxa belonging to 69 families and one unidentified local taxon. Dominant families were Asteraceae (14%), Solanaceae (7%), Rosaceae (6%), and Apiaceae (5%). The majority of use reports were attributed to the treatment of four disease categories: digestive (24%), skin (22%), respiratory (11%), and general (11%). The small overlapping portion (14 taxa mentioned by all three authors and another 27 taxa named by two authors) contained a high proportion of taxa (46%) mentioned in Dioscorides, which were widespread during that period in scholarly practice. Despite the shared flora, geographical vicinity, and culturally similar backgrounds, the medicinal use of plants in historical Courland and Livonia showed high biocultural diversity and reliance on wild taxa. We encourage researchers to study and re-evaluate the historical ethnobotanical literature and provide some suggestions on how to do this effectively.

## 1. Introduction

Historical ethnobotanical research has recently become an area of growing importance for researchers. The analysis of such data provides the grounds for a better understanding of how the Local Ecological Knowledge (LEK) of societies evolved over time, how these societies have used (local) plants, and how they have interacted with the environment and its components [1,2,3,4]. Nevertheless, comparing historical and contemporary data is not as easy as it may appear because of changes in the social, cultural, and socio-economic conditions of the studied societies [5]. However, investigators should continue the current trend of systemizing historical data collected by representatives of diverse scientific fields and extract the local ecological knowledge for future analysis [6,7].

Historical texts on plant use date back to around 3100 BC. One text from the common era is “*De Materia Medica*” [8], written by Greek physician Pedanius Dioscorides (AD 40–90), which inspired the medicine of that period and influenced many herbals published in the second half of the second millennium and especially earlier in the Middle Ages. During that period, herbal texts and recipe books were used as a standard means of creating knowledge about the medicinal usages of plants available. In the 19th century, inspired by Swedish botanist Carl von Linné (1707–1778), the gathering of local ethnomedicinal knowledge became popular, especially in the Nordic hemisphere. Such collections were also sometimes analyzed and published, inspiring future ethnobotanical research [6,9,10,11]. Such books, in addition to doctors and pharmacies, were important resources for the literate population for acquiring knowledge of simpler and more affordable medicines, and they contained, among local uses, traces of Dioscorides’s work. Leonti et al. [12,13,14] clearly demonstrated that in Italy the influence of the doctor and naturalist Pietro Andrea Mattioli’s (1501–1577) work is still apparent in studies from the last few decades: up to 20% can be traced back to Mattioli, and thus also, Dioscorides [14].

Although historical materials may be of crucial significance for understanding the evolution of local ecological knowledge, special attention needs to be given to the background and conditions in which the works were complied. Sōukand et al. [15] presented an example of a specific taxon (*Epilobium angustifolium*) that through confusion created by the existence of multiple concurrent names for the same species, incorrect translations, and illustrations supporting the transfer of usages from other species, led to a chain of misunderstandings and misinterpretations. In addition, not only the nomenclature of plants, but also disease names, change over time and specific knowledge is needed for interpretation. Therefore, special care needs to be taken in analyzing historical publications.

### 1.1. Background

While digitizing the German-language literature containing Local Ecological Knowledge of the 19th century for a database [16], as a practical part of the Master’s thesis of the second author [17], we selected three scientifically sound and valuable ethnobotanical works published in German on neighboring Livonia and Courland [18,19,20], which we consider to be the first summary studies on folk herbal medicine in that region.

Dr. Johann Wilhelm Ludwig von Luce’s (1756–1842) book “*Heilmittel der Ehsten auf der Insel Oesel*” (*Remedies of the Estonians of Oesel Island*), which was issued in 1829 in Pernau (Pärnu) [18], presents an original study on the local ethnomedicine of the island (currently Saaremaa). Having worked on the island for 38 years, first as a pastor and later as a doctor, Luce presented on 159 pages his own experiments as well as the local ethnomedical knowledge. Although his earlier book (in 1823) “*Topographische Nachrichten von der Insel Oesel, in medicinischer und ökonomischer Hinsicht*” (*Topographical News from the Island of Oesel, in Medical and Economic Terms*) [21] already contained the plant uses described in his earlier publication, we decided to focus our analysis only on the later publication as it is more voluminous.

Luce came to Saaremaa from Germany in 1781 as a pastor. After the tragic death of his wife, he returned to Germany to study medicine in 1789–1792. In 1801, he defended his doctor’s exam in St. Petersburg and after that became a practicing doctor in Saaremaa. His knowledge of botany was profound and his contribution to the scientific discipline is acknowledged by the presence of his own botanical author abbreviation. He wrote his book in order to share his experiences with local ecological knowledge, making it available to a wider audience [18,22]. He divided his book into chapters covering mineral, herbal (including pharmaceuticals herbs), animal, instrumental (also bloodletting, steam baths, and massage), and fantastic (magical rituals) components of respective remedies. All the reports were gathered almost exclusively on Oesel, although it has often been cited as reflecting data covering the whole of Estonia.

Jewish doctor Emil Aronson’s (1863–1942) article “*Ueber die Volksheilmittel der Letten*” (*On the Folk Remedies of the Latvians*), which was issued in 1891, is a 19-page-long contribution to the 19th volume of the journal “*Magazin lettisch-literarischen Gesellschaft*” (*Magazine of the Latvian Literature Society*) [19]. He explained the need for his work by citing the lack of Latvian data outlined in the doctoral dissertation of Dr. Wassily Demitsch (Bacилий Φeдopoвич Дeмич) (1858–1930), “*Literärische Studien über die wichtigsten russischen Volksheilmittel aus dem Pflanzenreiche*” (*Literary Studies about the Most Important Russian Folk Remedies from the Plant Kingdom*), which the latter defended at the University of Dorpat (Tartu) in 1888 [23]. Aronson studied medicine at the same university. In addition, he followed the structure of Luce’s book: sorted his own article by type of medicine, categorizing them into mineral, herbal, animal constituents, and applications. The data presented by Aronson originated almost exclusively from Libau (currently Liepāja), where his own doctor’s office was located, although he presented the data as filling the gap for Latvia in general. Aronson sometimes compared his results with those of Luce and Demitsch or referred to them for additional information.

In the foreword to his work, Aronson acknowledged the importance of documenting lay uses without any prejudice or contempt. Aronson wanted to show that, although most usages are superstition- or curiosity-driven, some could still be useful for contemporary medicinal science, and thus, there is the need to identify the good ones. He considered local medicines affordable and obtainable by everyone, while their effectiveness was supported by local beliefs and culture. Aronson did not provide any local names for plants or diseases. In 1893, he relocated to Dallas, USA, where he became a pioneer in public health.

Latvian medical student Jēkabs Alksnis’s (1870–1957) article “*Materialien zur lettischen Volksmedizin*” (*Materials on Latvian Folk Medicine*) [20] was issued in 1894 in the fourth yearbook of the University of Dorpat (Tartu), “*Historische Studien aus dem pharmakologischen Institute der kaiserlichen Universität Dorpat*” (*Historical Studies from the Pharmacological Institute of the Imperial University of Dorpat*). Alksnis studied in Tartu from 1890 to 1895. In the preface of the article, he mentions that he started this work at the request of Professor Eduard Rudolf Kobert (1854–1918), who also instructed and advised him. Alksnis apologizes for leaving a lot of material out of this work because of the length limit of the article, which was 117 pages long. It was mainly a summary translation into German of information from Latvian and Russian sources. For example, he translated with the permission of folklorist and poet Fricis Brīvzemnieks [Fricis Treilands] (1846–1907), a chapter on incantations from a book issued in 1881 in the Russian language “*Tpyды Этнoгpaφичecкoгo oтдeлa, Maтepиaлы пo этнoгpaφии лaтышcкoгo плeмeни*” (*Proceedings of the Ethnographic Department, Materials on the Ethnography of the Latvian Tribe*). In addition, he used many newspapers, such as the supplement newspaper “*Dienas Lapa*”, which published Latvian ethnographic writings. In the same newspaper, he published a call in 1892 for the collection of folk medicine and provided recommendations on how to collect data correctly [24]. Alksnis added many of his own experiences and mentions the names of other doctors, one sent him dried plant samples (Dr. P. Kalniņš) and another helped him to describe folk diseases (Dr. Raphael). The plants sent by Dr. P. Kalniņš were identified later by a botanist named Dr. Johannes Christoph Klinge. Alksnis also used folk medicine material from the Riga Latvian Society, which was sent to the society or collected by members of the society themselves.

It was not until 1898, however, that Alksnis published previously unpublished materials held by the Riga Latvian Society [25]. However, we have not analyzed this data in the present work, since at that time, general research on Latvian folk medicine became more active, and many similar articles began to appear in Latvian. One of these was written by the first Latvian botanist, Jānis Ilsters (1851–1889), who highlighted the use of Latvian folk medicines and folk plant names. His book contains descriptions of plants, yet lacks information about the source, clearly presenting facts from other countries [26]. In addition, he published several appeals to the public to help in reporting plant names and their application in folk medicine [27], but the data he collected were published posthumously in 1891 [28]. However, Ilster’s article is not included in the literature cited at the end of Alksnis’s article. Also, Riga pharmacist Ernests Birzmanis (Birsmanis) (1860–1900) published a call in 1897 in the newspaper “*Latweeschu Awises*” for the collection of folk medicine to gather information about treatments using plants. However, unlike Alksnis, he also paid attention to folk plant names [29]. Furthermore, he published one of the first Latvian-language books on Latvian medicinal plants [30], in which he indicated the most common folk plant names. He, too, studied at the University of Dorpat (Tartu), where he graduated in 1892 with a Master’s degree in pharmacy.

Alksnis outlines the scientific goals of his work as translating existing knowledge from Russian, educating Latvian doctors on local ethnomedicine and promoting the rationalization of drug administration, reflecting the scientific approach in the way his article is organized: background information on a disease is complimented by details on healing practices, drug preparations, and components. He provides local names only for the diseases, and not for the plants. Alksnis’s article can be seen as representative, as it covered the entire area inhabited by Latvians in Livonia and Courland. He later worked as a surgeon and was a professor of medicine at the University of Latvia from 1924 to 1944, emigrating to Germany during World War II and after the war to the UK. Today, the work of Alksnis holds great value for Latvian folk medicine history [31].

### 1.2. Aims of the Work

Dr. Wassily Demitsch’s doctoral dissertation was the first scientific study of plants used in folk medicine to summarize areas of Russia. However, as he stated, he reported only a small part of his work, describing only the most popular plants (he had over 65 species on the list). He says that there was a great deal of overlap with ancient Greco-Roman plant uses [23]. Other authors have suggested that there is a high degree of overlap with Hippocrates and Dioscorides in Russian territories [32]. At the same time, Demitsch stressed that doctors with an academic education did not care about or evaluate folk medicine and only relied on active ingredient-based (very expensive) treatments. However, people have prejudices and beliefs that prevent new therapies from being accepted by them. Demitsch notes that it is the cultural background of the community that could help the doctor to better explain to the people what is rational and what is not rational. A few years later, Leopold Glück (1854–1907), a Polish physician and public figure of Jewish descent, also emphasized the need to study the non-rational methods of folk medicine [33]. Thus, at that time, the contribution of general practitioner doctors to the study of folk medicine was significant.

What was also decisive for our choice was the fact that Luce, Aronson, and Alksnis shared a feature in common: they were doctors or medical students. As they lived during the same century and within close proximity to each other, the analysis of their work provides grounds for a detailed comparison. This allows shedding light on the biocultural diversity related to ethnomedicine and provide comparative data for field studies from the region.

The aims of this study are (a) to update and reconcile with current knowledge the identification of plants and diseases, identifying potential mistakes in the initial sources; (b) to compare the local plant uses described by the three authors; and (c) to evaluate the diversity of the sources. We expect to find high biocultural diversity in the three published sources.

## 2. Results

### 2.1. Disease and Plant Identification

The majority of all medical conditions described in the books could be assigned to specific modern disease categories. There were, however, some exceptions; for example, we assigned *artheibisches Fieber* mentioned by Luce solely to the general fever category, while *rose* mentioned by Alksnis can generally be identified as a skin disease, as there are various types of *rose* according to Luce. Likewise, the symptom *sich verhoben haben/sich verrissen haben/Verreissung*, attributed to “working too hard or with the wrong posture” [18,20], is a common condition described in Estonian folklore [34]. Not having more details to rely on, we interpreted it as indifferent back pain (musculoskeletal disease category).

Plant identification was sometimes a challenge, even though most of the time the authors provided the Latin name of the plant. One such example for Luce is *Cnicus serratuloides*, which is *Cirsium serratuloides* according to Plants of the World Online (https://powo.science.kew.org/, accessed on 7 March 2022). However, it was absent from the local floras, as the study area is outside of its natural range. The Estonian local name did not provide further clarity, and therefore, plant identification was kept at the genus level: *Cirsium* sp. Another example is *Ononis repens*, which was identified instead of *Ononis spinosa*, as given by Luce: *O. spinosa* does not grow in Saaremaa and according to a book of Estonian folk plant names [35], the term *luuderohi* is associated with *O. repens* in the Püha parish in Saaremaa. There are also mistakes outside the same genus, for example: *Hippocrepis comosa* was re-identified as *Argentina anserina*, as this taxon does not grow in Saaremaa and it is a clear misidentification by Luce, since the two taxa are visually very similar, while the name *hoolmerohi* was widespread in Kihelkonna parish in Saaremaa for *Argentina anserina* according to the book of Estonian folk plant names [35]. A difficult case of identification was a plant identified by Luce as *Equisetum fragile*, as such a name does not exist; however, its German name is *Engelsüß*, local name *rinna rohhi*, and it was used to treat a cough referred to as *Polypodium vulgare*.

The book by Alksnis also contained cases of difficult identification, like *Lappa* and *Lappa tournefortii*. While *Lappa* could refer to *Arctium lappa*, the most common taxon in the region is *Arctium tomentosum*, and the two are not differentiated on the popular level. Thus, we assigned both records to *Arctium* spp. *Thymus chamaedrys* is a wild thyme species in Western Europe, but it does not grow wild in modern-day Latvia, and thus it was re-identified as the local wild thyme *Thymus serpyllum*.

The only instance in which Alksnis confesses to having failed to identify a plant is described as follows: The Latvian people have a disease which they call the “suffocating” or the “choking” (speedejs un schnaudsejs). It is very bad: the sick roll on their beds and tear their hair out in despair “pinched”. From this description it follows that we are dealing with colic here. A herb called “speedeja sahle” (i.e., herb against the colic) is said to be very effective against this condition, its Latin name I have not yet been able to determine. [20] (pp. 191–192). We list the plant in our table as an unidentified taxon, but do not consider it in other analyses, unless explicitly named.

Two other taxa in Alksnis’s records needed special attention. *Sedum vulgare* is absent in the studied local floras [36,37,38] and has a single record in Estonia from 1864, the other records only come from Central Europe. Similarly, *Aconitum lycoctonum* has only a few records [37,39]. However, it is not similar enough to the local widespread taxon *Aconitum napellus*, which has blue flowers. Therefore, we identified the two taxa as *Sedum* sp. and *Aconitum* sp., respectively.

Alksnis in particular, but the other two authors as well, sometimes provided only the German name of some household cultivars like *Linde* (lime tree—*Tilia* spp.), *Kohl* (cabbage—*Brassica oleracea* L.), *Pflaumensaft* (plume juice), and *Turmkraut* (tower mustard—*Turritis glabra* L.). Cabbage was identified on the basis of the way in which it was prepared (fermented), as *Brassica oleraceae* L. was the only possible species that was prepared in that way at the time.

We identified some taxa solely to the genus level, which were also not differentiated on the popular level; for example, there are two taxa of *Tilia* growing in the area, of these *Tilia cordata* was most likely used, yet *T. latifolia* is also common, especially in cultivation. Of the four possible *Betula* species growing in the region, the ones most likely used were *B. pendula* and *B. pubescens*.

### 2.2. Overview of the Reported Taxa and Comparison between the Three Authors

**Figure 1:**
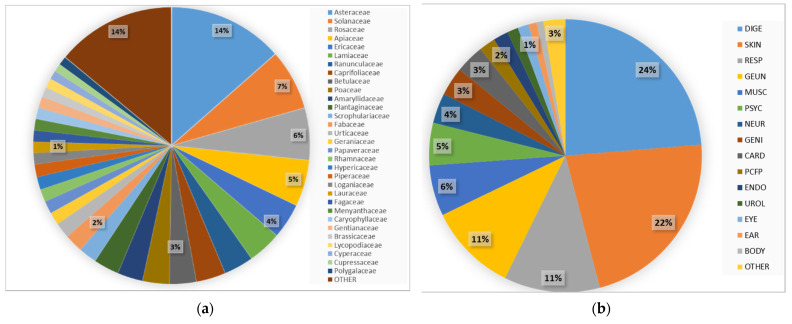
Proportional distribution of DUR among (**a**) plant families and (**b**) general disease categories. For abbreviations of the diseases see Table 1 below.

In total, there were 603 detailed reports of medicinal plant use, which refer to 219 taxa belonging to 69 families and one unidentified local taxon (Table 1). The dominant families were Asteraceae (14%), Solonaceae (7%), Rosaceae (6%), and Apiaceae (5%) (Figure 1). The majority of Detailed Use Report (DUR) was attributed to the treatment of four disease categories: digestive (24%), skin (22%), respiratory (11%), and general (11%).

The proportion of single DUR is high in the three studies: 50% for Alksnis and Aronson and 60% for Luce. The records with more than two DURs constitute 16% in the works of Luce and Aronson and 28% in Alksnis. Examples of multifunctionality include *Artemisia absinthium,* for which Luce reported five DUR, Alksnis reported six DUR, and Aronson reported two DUR; *Valeriana officinalis*, for which Luce reported two DUR, Alksnis reported eight DUR, and Aronson reported six DUR; and *Taraxacum officinale* (one DUR in all works).

Of all the taxa described by the authors, 22 were clearly purchased (12 of them described only by Alksnis). There is a high number of taxa described as wild by Friebe [40], but according to current classifications they are regarded as cultivated.

**Figure 2:**
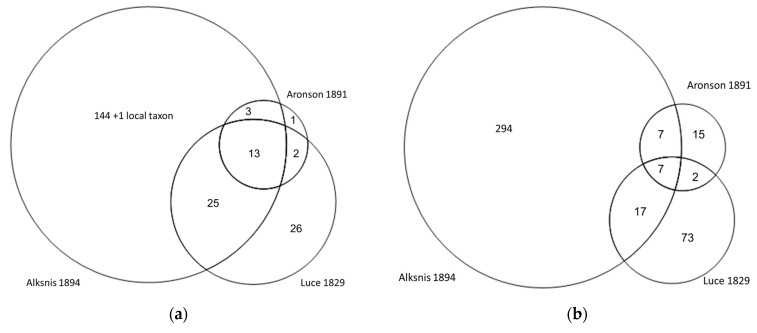
Proportional Venn diagrams showing the number of overlapping species (**a**) and Use Instances (UI) (**b**) among the three authors.

Alksnis’s article contained the greatest number of taxa, while he also had the highest number of taxa identified on the genus level (Figure 2, Table 2). Thirteen taxa were present in all three works: *Achillea millefolium*, *Allium cepa*, *Arnica montana*, *Artemisia absinthium*, *Betula*, *Levisticum officinale*, *Matricaria chamomilla*, *Nicotiana rustica*, *Strychnos nux*-*vomica*, *Taraxacum officinale*, *Valeriana officinalis*, *Verbascum thapsus,* and *Quercus robur*. Although Alksnis clearly used Aronson’s work (often referring to him), he did not include three taxa (*Cinnamomum camphora* shared with Luce and *Prunus cerasus* and *Ferula assa-foetida* mentioned solely by Aronson—of them only *Prunus cerasus* grows locally).

While for all three authors the Asteraceae family had the most mentioned taxa and DUR, the other botanical families differ. In Luce’s work, the second and third most important families are Ranunculaceae and Apiaceae, while in Alksnis, they are Solanaceae and Rosaceae and in Aronson they are Caprifoliaceae and Lauraceae. In terms of species, Luce recorded the highest diversity of general categories in which the plant is used for *Ranunculus acris* and *Hypericum perforatum*; Alksnis for *Arnica montana*, *Juniperus communis*, *Urtica urens,* and *Hyoscyamus niger*; and Aronson for *Veronica officinalis* and *Matricaria chamomilla*. The number of DUR per plant varies from author to author.

Skin and digestive diseases are the most mentioned medicinal use categories for all three authors, and while general and unspecified diseases are the third most mentioned category for Luce and Aronson, they are number four for Alksnis. For Alksnis, respiratory diseases represent the third most-mentioned category, whereas the other two authors reported far fewer DUR in this category (Table 2).

The Jaccard Similarity Indexes show the highest overlap between Luce and Aronson on both the taxa and use instance (UI) level (Table 3). While there are some similar uses mentioned by all three, or at least two, of the authors, there are many more different applications. *Artemisia absinthium*, being the most diversely used plant, shows some overlaps (dysentery and abdominalgia in Luce and Alksnis, fever in Alksnis and Aroson) as well as divergences in use (ulcers and malaria in Luce, and internal diseases and actual neurosis in Alksnis). *Tanacetum vulgare* is a rather rare example of complete agreement in use: worm infestation in both Luce and Alksnis.

An interesting example is that of *Rhododendron tomentosum*, used to treat lice in Luce, to which Alksnis added uses against pulmonary tuberculosis, bone pain, and general deteriorating health. Luce reported the use of *Ranunculus acris* against gout, dropsy, vesicating, amaurosis, hip pain, rheumatism, and fever, while Alksnis reported its use to treat cold and burn wounds. Likewise, Luce mentioned the use of *Viola tricolor* against skin diseases, whereas Alksnis noted it use against whooping cough. While Luce recorded the use of *Silene vulgaris* against urological diseases, Alksnis mentioned its use to treat joint rheumatism (musculoskeletal category).

## 3. Discussion

### 3.1. Why Such Diversity?

The Jaccard Similarity Index is remarkably low: for comparison, the lowest JI from the region recorded in recent years was over 0.54 for all taxa. As the highest similarity was evident between the authors providing the fewest plants and uses, the size of the collection and the region covered play a significant role: while Luce collected on a small island, Aronson covered part of the mainland, which was about three times larger. Considering that the distance between the two regions was around 100 km over the sea and the time between collection dates was about 60 years (two generations), the difference is still considerable. Notably, both authors relied on long-standing personal experiences from the region in which they worked and their own discoveries. Another aspect to consider concerns the plant identifications made by the authors.

While the work of Aronson received little attention from detractors, Luce’s work was criticized by some of his contemporaries. Schmidt [39] complained that Luce’s works were not reliable, while Lehmann [41] accused him of listing “dubious species” that were not taken into account by later botanists. Regardless of such observations, the majority of later authors cite Luce (often referring to the whole of Estonia).

Aronson and Alksnis published during the same decade, yet the methodology of Alksnis was very different. Remarkably, Alksnis used Luce’s and Aronson’s works, but he did not copy them, instead he seems to have used them as a reference for a similar use. As Alksnis covered all of Livonia and Courland, he did not obtain the data by practicing there. Moreover, Alksnis’s age (at the time of publishing his article he was just 24 years old) did not allow for him to accumulate much practical experience. In addition to his own collection, Alksnis relied on the data mediated by the newspaper and this aspect was heavily criticized by his contemporary colleague, pharmacist Ernests Birzmanis [42]. Birzmanis pointed out the lack of original (Latvian) names in the descriptions of medicinal remedies, questioning the ability to substantiate the accuracy of the Latin and German translations. In the preference to his work, Birzmanis emphasized that with the given report he did not want to diminish the value of Alksnis’s published book, but rather with his notes help subsequent authors in the field to correct mistakes in their respective books of interest. Birzmanis noticed several errors in the translations for both taxa and illnesses; however, we cannot automatically consider those adequate. For example, while Alksnis mentions the use of *Artemisia vulgaris* bulbs for treating nerve diseases, Birzmanis emphasizes that “viboksne” (“vibotne”), which corresponds to *Artemisia vulgaris*, does not have bulbs and thus the translation of the Latvian name must have been incorrect. Additionally, Alksnis mentions “kruklis”—for which his book offers three different taxa having this name, while Birzmanis presents *Rhamnus frangula* as the corresponding one. Those two mistakes are probably the most serious additions to the text, yet as the exact source of the information cannot be verified, we decided to keep it unchanged. Birzmanis also criticizes German translations; however, here it is impossible to confirm them. For example, “pirts slota” should have been translated as “die Quast” not “der Basen”. As for illnesses, Birzmanis notes “meris” should have been recorded as “die Pest” not “die Seuche” and adds that such translation errors can be found in several places. Birzmanis also adds that in many places the medicinal applications are quite disgusting and thus he doubts these uses are common, likely being utilized by only one or two strange individuals; and if this is in fact the case, he believes they cannot be considered part of Latvian folk medicine, thus trying to idealize Latvian medicine. For example, the use of “cukas zults ar lapu tabaku” (pig bile with tobacco leaves) to treat swellings corresponds to a single event and thus does not warrant inclusion in Latvian folk medicine. However, Birzmanis adds that for the materials, Alksnis himself collected or borrowed from Fricis Brīvzemnieks’s book—such silly things are not found. Birzmanis points out that for the materials referred to by Dr. Raphael, the information seems miraculous and unbelievable [42].

Relying on current knowledge, we have observed that the authors, especially Luce, made several potential identification errors leading to the recording of taxa that do not grow in the region. Therefore, regardless of all our efforts to minimize errors and misinterpretations, we cannot guarantee that all the initial identification mistakes have been eliminated. This needs to be taken into consideration while working with the data.

Works by contemporaries of Luce support the idea of the diversity of ethnomedicine at that time. The closest in time to Luce was the unpublished manuscript of pastor and amateur botanist Johann Heinrich Rosenplänter (1782–1846), along with his field notes and loose-leaf herbarium vouchers, which have been thoroughly analyzed recently, showing just a few overlapping plants and no overlap on the use level with Luce’s work originating from the same decade (1820–1830) [43].

Another comparable work is that of Dr. Mihkel Ostrov (1863–1940), who not only studied medicine at the University of Dorpat at almost the same time as Alksnis, but was also interested in folk medicine. Ostrov collected medicinal plant knowledge from across Estonia through an appeal in the newspaper. Unlike Alknis, Ostrov also placed great emphasis on the popular names of medicinal plants. Comparing Ostrov’s data provided in the manuscript with the article in Alksnis, we found that nearly two-thirds of the used plants overlapped, whereas the uses differed in majority of cases [44]. However, the study of Ostrov was not complete, so the comparison is not fully informative.

Some more similarities can be seen on the genus level with the results obtained by Sile et al. [45], but as their methodology and the actual time of collection of the folklore which is the basis for their study is not stated, a more detailed comparison is not feasible. Moreover, as Alksnis and several other authors also later published in Latvian, the later folklore might have been influenced by literary sources and cannot be considered a good base for comparison.

Therefore, we can assume, regardless of any possibly remaining mistakes in identification, that plant use in the 19th century in the region was highly diverse and place-specific. Despite the criticism of Birzmanis towards presenting singular uses, we should not underestimate the importance of even singular uses of local wild taxa, which represent part of the local biocultural diversity. In this framework, for understanding the patterns of formation of LEK, we need to differentiate between locally developed and global, introduced knowledge, which may have already become local. Therefore, source criticism, taking into account the high possibility of error even in the interpreted text or data, as well as in the possible tracking of the origin of the data, is necessary [12,46].

The selected works are situated in very similar local environments and the diseases treated in the 19th century were quite similar. However, comparing the numbers of plants and uses each author provided, we also need to keep in mind the size of the region the authors covered, which can contribute to the perceived differences in the taxa use. Nevertheless, as we see high divergence in uses regardless of the limits set by flora and diseases, we can assert that an interchange of LEK within the 19th century only occurred for a limited number of species, then related to official medicine. This is very different from the current situation in the region, where overlap is very high and clear signs of homogenization caused by official medicine of the Soviet era can be seen [47].

### 3.2. Comparison with Dioscorides

**Figure 3:**
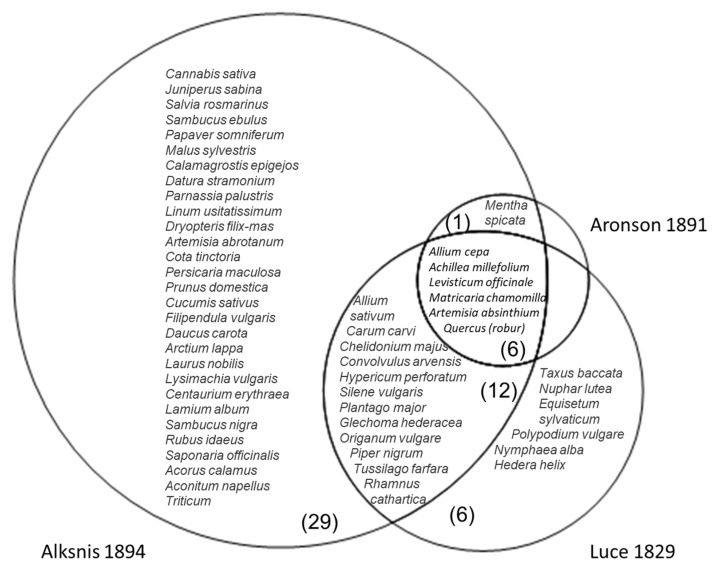
Taxa represented in the analyzed books and Dioscorides.

Overlap with the taxa mentioned in Dioscorides’s *Materia medica* differs among the authors, being highest for Aronson (39%), followed by Luce (38%) and then Alksnis (26%) (Figure 3). However, the highest percentage of overlap (47.5%) is found among the taxa that overlap between the authors (either all three or two of them), while the remaining taxa represent 25% for Luce and 21% for Alksnis. In all cases, the percentage is higher than the 20% proposed by Leonti et al. [14].

Looking at the species combined with their medicinal use, commonly mentioned taxa include *Artemisia absinthium* (digestive and general and unspecified), *Carum carvi* (digestive), *Plantago major* L. (skin), *Chelidonium majus* (skin), and *Achillea millefolium* (skin). Frequently mentioned usages of species are mostly from the medicinal use categories with the most DUR, such as skin, digestive, respiratory, general, and unspecified diseases and symptoms.

Such overlap suggests a potential, although not direct, influence of Dioscorides on local ethnomedicine. However, we need to take into account that both Luce and Aronson had a lot of practical experience and developed remedies on their own, thus not only influencing local ethnomedicine, but also perhaps including their own knowledge without explicitly acknowledging it, creating a “feedback loop” as described by Leonti [12]. This can, in fact, cause an overestimation of the impact of Dioscorides.

Another important point is that the influence of herbals and books on plants and folk medicine should be evaluated along with the literacy level of a society. In Livonia, the study area of this paper, the “reading revolution” did not take place until the middle of the 19th century [48]. Poorly literate people were not able to contribute to, understand, or have access to the increasing numbers of books and texts, but needed mediators able to transmit such knowledge. While we acknowledge that filtering from scientific publications to common use is a known phenomenon in society [49], we need to understand how such book-based knowledge transferred into practice.

We also cannot underestimate the potential self-discovery of some uses (even if they overlap with Dioscorides). A good example is provided by *Carum carvi*: peasants had an obligation to collect the seeds for the manors, and therefore, they were familiar with the plant and always had it available and used it for food [50]; as a result, many ad hoc uses could also have been tried on demand and entered into circulation after yielding positive results. Another example may be that of *Arnica monatna*. The first Latvian herbal book [42] says that *Arnica montana* grows in Courland, but this claim was later refuted. At that time, many similar yellow-flowered local species could have been mistakenly identified as *A. montana*, as happened among Estonians living in Livonia [51].

### 3.3. Aspects to Consider When Interpreting the Data

The increasing influence of pharmacies and doctors in the 19th century influenced people in their wild plant usage. Although this has an effect on people’s medicinal plant usage, i.e., certain usages decrease or disappear as a result of switching to pharmaceutical products, this influence is, in retrospect, difficult to quantify, especially because other factors like people’s beliefs in certain cultural treatments, as well as an aversion to pharmacies and doctors, also have to be considered here.

**Figure 4:**
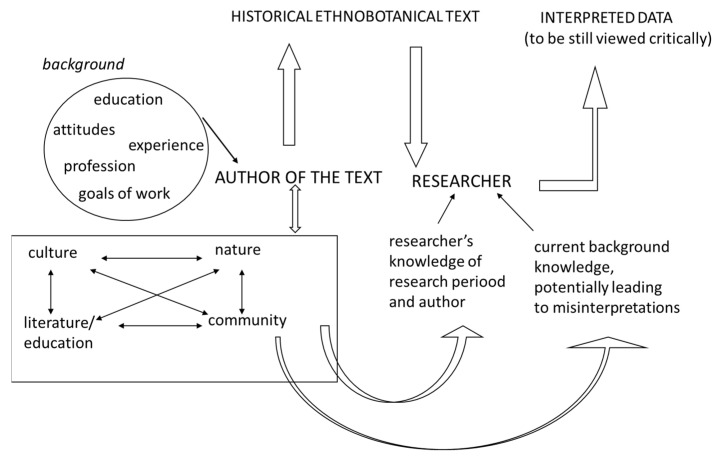
Aspects to consider when interpreting historical ethnobotanical works. Adapted from Sõukand [52].

We recommend that future researchers wanting to interpret historical sources consider the following aspects (summarized in Figure 4):

(1) The greatest possible attention needs to be given to the background of the authors and the general context in which the work was written, the available supporting tools, and the existing knowledge of that time.
(2) It is important to understand the metalanguage of the source, e.g., one needs to study the source in the context of the time it was written and understand the natural, cultural, and societal settings of that time. This not only applies to historical archival sources, but also to earlier ethnobotanical literature.
(3) Keep in mind that current background knowledge (on nature, culture, society, literature, etc.) can influence the interpretation of historical data, potentially leading to misinterpretations as the researcher may involuntarily assume the contexts have some elements in common.
(4) Throughout all the work, and also after the interpretation is completed, vigilant source-criticism and self-criticism need to be present.

## 4. Materials and Methods

An Excel database was created by manually selecting relevant information and entering it into the database. Every independent use in the sources was accounted for as a Detailed Use Report (DUR), where the informant *i* mentions a specific medicinal use, based on the use categories of the specific author, of the plant part (*p*, e.g., fruits, leaves, aerial parts, flowers, etc., if provided), considering also the form in which the plant part is used (*f*, e.g., fresh, dried, frozen, refrigerated) and specific way of preparation, if provided. Every DUR was entered on a separate row in the excel spreadsheet.

The historical medicinal data (originally mentioned disease or symptom recorded in German) was interpreted on the basis of the provided name and its correspondence to equivalents reported in historical and current literature [53,54]. To identify general disease categories, we relied on the symptoms or conditions associated with the disease and the ICPC-2 classification [55] was applied for comparative purposes. For comparison, we also calculated Use Instances (UI), where one UI corresponds to the specific plants used in an etic disease category according to the ICPC-2, regardless of the number of different emic diseases treated. Comparison between the three ethnobotanical sources was made using the Jaccard Similarity Index (JI), adopting the methodology of González-Tejero et al. [56]: JI = (C/(A+B − C)), where A represents the number of taxa/UI in sample A, B is the number of taxa/UI in sample B, and C is the number of taxa/UI common to A and B.

The plants and their names given in the books were checked for reliability using:

- Flora Europaea [57] to verify the plant identifications and with floras of that time [36,37,38] to confirm that the plants really grew in the region when the books were published;
- Vilbaste [35], Beiche [58], Genaust [59], and Hiller and Melzig [60] comparing the local and German names and descriptions;
- online biodiversity databases (https://elurikkus.ee/ and https://www.latvijasdaba.lv/, accessed on 29 October 2021) to confirm the presence and distribution of the taxa in the region;
- other herbal texts and books [61,62,63].

The current names provided follow Plants of the World Online [64], except for two taxa (*Taraxacum officinale* and *Ononis repens*), which are based on Flora Europaea [57].

Graphs and diagrams were created with Excel, while proportional Venn diagrams were created using the PAST Toolkit Venn diagram plotter software program (https://omics.pnl.gov/software/venn-diagram-plotter, accessed on 26 October 2021).

### Region

**Figure 5:**
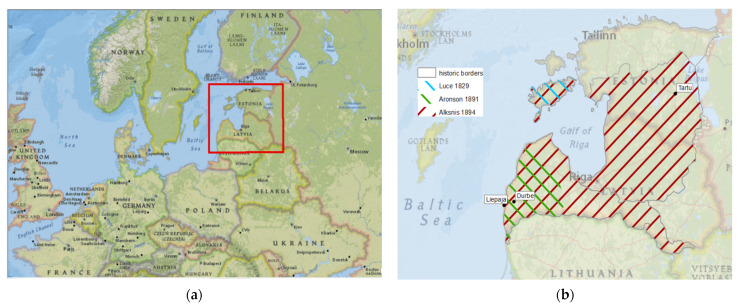
(**a**) Study region within Europe; (**b**) regions where authors worked (ArcGIS, historical borders according to H. Laakmann [65], edited by the second author M.A.).

The area of data gathering for Alksnis included present-day Latvia and southern Estonia, while Luce covered modern-day Saaremaa and Aronson, which are the surroundings of present-day Liepaja (Figure 5).

Latvia’s and Estonia’s landscapes belong to the Eastern European hilly lowlands (the highest point being 318 m above sea level), and consist of approximately 50% (mainly) pine forest, alternating with meadows and swamps and mixed deciduous forest towards the south. A wide variety of wild berries and mushrooms also grow there [66], see also Lehmann [41] and Schmidt [39]. The climate in Estonia and Latvia is similar: bordered by the Baltic Sea, it is moist and humid with an annual precipitation between 600 and 700 mm. Podzol soils predominate [66].

Livonia and Courland underwent constant changes in rulership and repeated attempts at Germanization and Russification, heavily affecting the diverse local cultures. The first invasion started in the 13th century and German influence lasted with various interruptions of leadership until World War I [65,67,68]. Divided into two at the end of the 16th century, Livonia was conquered by Sweden, while Courland remained with Poland; the 18th century saw the Russian occupation of both regions and the beginning of the 20th century brought independence [65,67,68,69,70].

During this time of continual leadership changes, the influence and importance of Germans and Baltic Germans remained the same and was even intensified by several waves of German immigration. Among the immigrants, there were often intellectuals and academics who were immediately accepted as part of the higher social classes. Another effect of this cultural influence and the immigration of academics was that the academic language in the region was German for a long time. Also, peasants, being mostly locals and making up the majority of inhabitants, remained illiterate for a long time. Therefore, the field of science in Livonia was established by Germans and Baltic Germans, and as a result, the German language was utilized up until the end of the 19th century [71,72,73].

## 5. Conclusions

Our results demonstrate high biocultural diversity in terms of taxa and their medicinal use within a limited temporal and spatial context, especially regarding the use of local, wildly growing plants. The high overlap among the three authors and with scholarly sources as well as the use of cultivated and purchased taxa do not diminish the value of the biocultural diversity of the medicinal use of locally growing plants. The authors encourage researchers to study and re-evaluate historical ethnobotanical works from recent centuries in Europe in order to better evaluate the evolution of medicinal ethnobotany.

## Figures and Tables

**Table 1:** Plants named by all three authors and general disease categories.

Family	Latin Name	Name in the Source	Local Name	Origin of Plant	Luce	Aronson	Alksnis
Acoraceae	*Acorus calamus* L.	*Acorus Calamus*		C, W ^F^			DIGE, GEUN, MUSC
Amaranthaceae	*Atriplex* sp.	*Atriplex*		W, C			SKIN
	*Beta vulgaris* L.	*Beta vulgaris*		C			DIGE, RESP
Amaryllidaceae	*Allium cepa* L.	*Allium cepa*	Sibbulas	C	GEUN	SKIN	GENI, PSYC, RESP, SKIN
	*Allium sativum* L.	*Allium sativum*	Küislauk	C	DIGE		EAR
	*Allium schoenoprasum* L.	*Allium schoenoprasum*		W			RESP
	*Alloideae* sp.	*Allioideae*		W			NEUR
Apiaceae	*Angelica archangelica* L.	*radix Angelicae*		C ^F^, P(?)			DIGE
	*Carum carvi* L.	*Carum carvi*	Köömled	W, C, P ^F^	DIGE, PCFP		DIGE, ENDO, RESP
	*Cicuta virosa* L.	*Cicuta virosa*		W ^F^			GEUN, NEUR, SKIN
	*Daucus carota* L.	*Daucus Carota*		C			DIGE
	*Ferula assa-foetida* L.	*Ferula asa foetida*, *Scorodosma foetidum*	Tiwistriik	P	CULT	DIGE	PSYC
	*Laserpitium latifolium* L.	*Laserpitium latifolium*		W			DIGE
	*Levisticum officinale* W.D.J.Koch	*Levisticum officinale*	Liibstocki rohhi	C ^F^	SKIN	GEUN	CARD GEUN MUSC NEUR
	*Petroselinum crispum* (Mill.) Fuss	*Petroselinum crispum*		C			DIGE, GEUN, SKIN, UROL
	*Peucedanum ostruthium* (L.) W.D.J.Koch	*Peucedanum ostruthium*, Radix Imperatoriae		P			DIGE
	*Pimpinella* sp.	*Pimpinella* L.		W	GEUN		CARD
Araliaceae	*Hedera helix* L.	*Hedera helix*	Ragga mailase rohhi, lude rohhi	W ^F^	MUSC, SKIN		
Asparagaceae	*Polygonatum odoratum* (Mill.) Druce	*Convallaria polygonatum*		W ^F^			MUSC
Asphodelaceae	*Aloe* sp.	*Aloe*		C			DIGE
Asteraceae	*Achillea millefolium* L.	*Achillea millefolium*	Raudrohhi	W, C ^F^	SKIN	RESP	BLIM, RESP, SKIN
	*Anthemis arvensis* L.	*Anthemis arvensis*		W			SKIN
	*Arctium* spp.	*Lappa*^AL^, *Lappa tournefortii*^AL^, *Arctium Lappa* L. ^D^		W ^F^			NEUR, SKIN
	*Arnica montana* L.	*Arnica montana*^L, AL^, not stated ^AR^	Ärratöstmisehaiguse rohhi	P, (W ^F^)	MUSC	GEUN, MUSC	DIGE, GEUN, MUSC, RESP, SKIN
	*Artemisia abrotanum* L.	*Artemisia abrotanum*		C ^F^			GENI, SKIN
	*Artemisia absinthium* L.	*Artemisia absinthium*^L^, *Artemisia Absynthium*^AL^, *Artemisia Absynthium*^AR^	Koi rohhi	C, (W ^F^)	DIGE, GEUN, SKIN	DIGE, GEUN	DIGE, GEUN, PSYC
	*Artemisia cina* Berg ex Poljakov	*Flores cinae*		P		DIGE	DIGE
	*Artemisia sieberi* Besser	*Artemisia sieberi*	Ussi rohhi	P	DIGE		
	*Artemisia vulgaris* L.	*Artemisia vulgaris*		W ^F^			NEUR
	*Calendula officinalis* L.	*Calendula officinalis*	Koltsed aja öied	C	GENI, RESP, SKIN		DIGE
	*Cirsium vulgare (Savi) Ten.*	*Cirsium lanceolatum*		W			RESP
	*Cota tinctoria* (L.) J.Gay	*Anthemis tinctoria*		W ^F^			DIGE
	*Centaurea cyanus* L.	*Centaurea cyanus*		W, C ^F^			EYE, PSYC, RESP, UROL
	*Helichrysum arenarium* (L.) Moench	*Helichrysum arenarium*		W			SKIN
	*Jacobaea vulgaris* Gaertn.	*Jacobaea vulgaris*	Rist hoolmete rohhi	W	GENI		
	*Leucanthemum vulgare* (Vaill.) Lam.	*Chrysanthemum Leucanthemum*		W			DIGE, SKIN
	*Matricaria chamomilla* L.	*Matricaria Chamomilla*^L^, not stated ^AL^	Kummelid	P, C ^F^	GEUN	EYE, PCFP, PSYC	CARD, DIGE, GEUN, PCFP
	*Solidago virgaurea* L.	*Solidago virgaurea*	Hoolmete rohhi	W ^F^	DIGE, SKIN		
	*Tanacetum vulgare* L.	*Tanacetum vulgare*	Reinware rohhi, solika rohhi	W, C ^F^	DIGE		DIGE
	*Taraxacum officinale* F.H.Wigg. (coll.)	*Taraxacum campylodes*^L^, *Leontodon Taraxacum*^AL, AR^	Sea öied, sea pima rohhi, sea nuppud, woi rosid	W ^F^	SKIN	SKIN	SKIN
	*Tussilago farfara* L.	*Tussilago farfara*	Paiso lehhed	W ^F^	SKIN		GEUN
Balsaminaceae	*Impatiens nolitangere* L.	*Impatiens tangere noli*		W ^F^			SKIN
Betulaceae	*Alnus glutinosa* (L.) Gaertn.	*Alnus glutinosa*		W			DIGE, SKIN
	*Betula* spp.	*Betula pubescens*^L^, *Betula alba*^AR^	Kasse pu	W, C ^F^	BLIM, DIGE	DIGE	DIGE, MUSC, PFCP, SKIN
Boraginaceae	*Myosotis* sp.	*Myosotis*		W			PSYC
	*Symphytum officinale* L.	*Symphytum officinale*		W ^F^			SKIN
Brassicaceae	*Armoracia rusticana* P.Gaertn., B.Mey. & Scherb.	*Cochlearia armoracia*		C ^F^			DIGE
	*Berteroa incana* (L.) DC.	*Berteroa incana*		W			PSYC
	*Brassica oleraceae* L.	Saures Kohlblatt, sauerer Kohl		C			NEUR
	*Cardamine pratensis* L.	*Cardamine*		W			CARD
	*Cochlearia officinalis* L.	*Cochlearia officinalis*		P			CARD
	*Raphanus raphanistrum* subsp. *sativus* (L.) Domin	*Raphanus niger*		W^F^			RESP, MUSC
Campanulaceae	*Campanula trachelium* L.	*Campanula trachelium*		W			GEUN
Cannabaceae	*Cannabis sativa* L.	*Cannabis sativa*		C, (W) ^F^			DIGE, RESP
	*Humulus lupulus* L.	*Humulus lupulus*	Hummalad	W, C ^F^	DIGE		
Caprifoliaceae	*Succisa pratensis* Moench	*Succisa pratensis*^L^, *Scabiosa succisa*^AL^	Tölbi jurega pibe lehhed, peetri pibe lehhed	W ^F^	DIGE, GEUN		DIGE
	*Valeriana officinalis* L.	*Valeriana officinalis*	Paldrian, üllekäija rohhi	W ^L^, W ^F^	DIGE, PFCP	DIGE, GENI, GEUN, NEUR	CARD, DIGE, PSYC, RESP
Caryophyllaceae	*Dianthus deltoides* L.	*Dianthus deltoides*		W			DIGE
	*Herniaria glabra* L.	*Herniaria glabra*	Söötreia rohhi	W	SKIN		
	*Saponaria officinalis* L.	*Saponaria officinalis*		W, C ^F^			PSYC, SKIN
	*Silene vulgaris* (Moench) Garcke	*Silene vulgaris*^L^, *Silene inflata*^AL^	Pöie rohhi	W	UROL		MUSC
	*Stellaria media* (L.) Vill.	*Stellaria media*		W			GEUN
Celastraceae	*Parnassia palustris* L.	*Parnassia palustris*		W			CARD, GEUN
Convolvulaceae	*Convolvulus arvensis* L.	*Convolvulus arvensis*^L^, *Convolvulus*^AL^	Jooksja rohhi, kurre katlad, lippo rohhud, lippo warrekad	W	ENDO		SKIN
Crassulaceae	*Sedum acre* L.	*Sedum acre*		W ^F^			GEUN, MUSC
	*Sempervivum globiferum* L.	*Sempervivum soboliferum*		W			EAR
Cucurbitaceae	*Cucumis sativus* L.	*Cucumis sativus* L.		C			DIGE
Cupressaceae	*Juniperus communis* L.	*Juniperus*		W, (C ^F^)			CARD, DIGE, EAR, RESP, SKIN
	*Juniperus sabina* L.	*Sabina*		W, (C) ^F^			PCFP
Cyperaceae	*Carex arenaria* L.	*Carex arenaria*		W ^F^			GENI, MUSC
	*Carex flava* L.	*Carex flava*		W ^F^			RESP
Equisetaceae	*Equisetum hyemale* L.	*Equisetum hyemale*		W ^F^			CARD, GENI
	*Equisetum* sp.	*Equisetum*		W ^F^			MUSC
	*Equisetum sylvaticum* L.	*Equisetum sylvaticum*	Rammi rohhi	W	DIGE		
Ericaceae	*Arctostaphylos uvaursi* (L.) Spreng.	*Arctostaphylos uvaursi*		W ^F^			DIGE
	*Chimaphila umbellata* (L.) Nutt.	*Chimaphila umbellata*		W			MUSC
	*Empetrum nigrum* L.	*Empetrum nigrum*		W, (C ^F^)			DIGE, SKIN
	*Rhododendron tomentosum* Harmaja	*Ledum palustre*	Käelud	W	SKIN		CARD, GEUN, MUSC, RESP, SKIN
	*Pyrola rotundifolia* L.	*Pyrola rotundifolia*	Lambakörwad, lutöbbi rohhi	W ^F^	ENDO		
	*Vaccinium oxycoccos* L.	*Vaccinium oxycoccus*		W ^F^			NEUR
	*Vaccinium myrtillus* L.	*Vaccinium myrtillus*		W ^F^			DIGE, RESP
	*Vaccinium vitisidaea* L.	*Vaccinium vitisidaea*		W ^F^			GEUN, MUSC, SKIN
Euphorbiaceae	*Euphorbia helioscopia* L.	*Euphorbia helioscopia*		W			DIGE
Fabaceae	*Cassia fistula* L.	*Cassia fistula*		P			RESP
	*Glycyrrhiza glabra* L.	*Glycyrrhiza glabra*	Kolne pu	P	GENI, MUSC		
	*Ononis repens* L.	*Ononis spinosa*	Lude rohhi	W ^F^	ENDO, MUSC		
	*Senna alexandrina* Mill.	*Foliae sennae*		P			DIGE
	*Trifolium aureum* Pollich	*Trifolium agrarium*		W ^F^			DIGE, GENI
Fagaceae	*Quercus infectoria* G.Olivier	*Quercus infectoria*		P			DIGE
	*Quercus robur* L.	*Quercus robur*	Tamme pu	W, C ^F^	SKIN	DIGE	DIGE, GEUN
Gentianaceae	*Centaurium erythraea* Rafn.	*Erythraea centaurium*		W ^F^			DIGE, MUSC
	*Gentiana* sp.	*Gentiana*		W			DIGE
	*Gentianella amarella* (L.) Harry Sm.	*Gentiana amarella*		W			DIGE, PSYC
Geraniaceae	*Erodium cicutarium* (L.) L’Hér.	*Erodium cicutarium*		W			DIGE
	*Geranium pusillum* L.	*Geranium pusillum*		W			RESP, SKIN
	*Geranium robertianum* L.	*Geranium robertianum*	Rülli küined, russekud, punnase rosi rohi	W ^F^	SKIN		
	*Geranium* sp.	*Geranium*		C			EAR
	*Geranium sylvaticum* L.	*Geranium sylvaticum*		W			GENI
Grossulariaceae	*Ribes rubrum* L.	*Ribes rubrum*		W, C ^F^			RESP
Hypericaceae	*Hypericum perforatum* L.	*Hypericum perforatum*	Emmaste rohhi, raeste punned	W ^F^	DIGE, GENI, RESP, SKIN		GENI, GEUN
Iridaceae	*Crocus* sp.	*Crocus*		C			DIGE
	*Gladiolus* sp.	*Gladiolus*		C			DIGE
Lamiaceae	*Glechoma hederacea* L.	*Glechoma hederacea*	Rosi rohhi, kassi naered	W ^F^	SKIN		DIGE, RESP
	*Lamium album* L.	*Lamium album*		W			GENI
	*Mentha × piperita* L.	*Mentha piperita*		C, (W) ^F^			CARD, DIGE, MUSC, NEUR, RESP
	*Mentha spicata* L.	*Mentha crispa*		C, (W) ^F^		NEUR	DIGE, RESP
	*Origanum vulgare* L.	*Origanum vulgare*	Naeste punned	W ^F^	GENI		DIGE
	*Prunella vulgaris* L.	*Prunella vulgaris*		W			RESP
	*Salvia rosmarinus* Spenn.	Rosmarinöl		C, P			NEUR
	*Salvia glutinosa* L.	*Salvia glutinosa*		C			GENI
	*Thymus serpyllum* L.	*Thymus chamaedrys* ^AL^ *Thymus serpyllum* ^L^	Rabanduse rohhi	W, (C) ^F^	SKIN		RESP
Lauraceae	*Cinnamomum camphora* (L.) J.Presl	*Cinnamomum camphora*^L^, *Laurus Camphora*^AR^	Kampwer	P	ENDO	DIGE, EAR, GEUN	
	*Laurus nobilis* L.	*Laurus nobilis*	Loorberid	P			GEUN, SKIN
Linaceae	*Linum catharticum* L.	*Linum catharticum*		W ^F^			PSYC
	*Linum usitatissimum* L.	*Linum usitatissimum*		C			EYE, GEUN, SKIN
Loganiaceae	*Strychnos nuxvomica* L.	*Strychnos nuxvomica*^L, AR^, *Nux vomica*^AL^	Rebbase rohhi	P	DIGE	DIGE, GEUN	DIGE, NEUR, SKIN
Lycopodiaceae	*Lycopodium clavatum* L.	*Lycopodium clavatum*	Nöia rohhi, terwise rohhi	W ^F^	DIGE, SKIN		
	*Huperzia selago* (L.) Bernh. ex Schrank & Mart.	*Lycopodium selago*		W ^F^			DIGE, SKIN
Malvaceae	*Tilia* sp.	Lindenblüthentee		W, C ^F^			DIGE, GEUN, RESP, SKIN
Melanthiaceae	*Paris quadrifolia* L.	*Paris quadrifolia*	Hora marjad, ussilak	W ^F^	GEUN		
Menyanthaceae	*Menyanthes trifoliata* L.	*Menyanthes trifoliata*		W ^F^			CARD, DIGE, GEUN, NEUR, RESP
Nymphaeaceae	*Nuphar lutea* (L.) Sm.	*Nuphar lutea*	Koltsed kuppo lehhed	W, (C) ^F^	CARD		
	*Nymphaea alba* L.	*Nymphaea alba*	Wallged kuppo lehhed	W ^F^	CARD		
	*Nymphaea* sp.	*Nymphaea*		W			GEUN
Oleaceae	*Fraxinus excelsior* L.	*Fraxinus excelsior*		W, C ^F^			MUSC, NEUR
	*Syringa vulgaris* L.	*Syringa*		W ^F^			RESP
Orchidaceae	*Dactylorhiza maculata* (L.) Soó	*Orchis maculata*		(W) ^F^			GENI, PCFP
	*Epipactis palustris* (L.) Crantz	*Epipactis palustris*		W			ENDO, PSYC
Orobanchaceae	*Pedicularis* sp.	*Pedicularis*		W			SKIN
Oxalidaceae	*Oxalis acetosella* L.	*Oxalis acetosella*		W ^F^			GEUN
Papaveraceae	*Chelidonium majus* L.	*Chelidonium majus*	Werre urma rohhi	W, C ^F^	DIGE, EYE, SKIN		SKIN
	*Papaver somniferum* L.	*Papaver somniferum*		W, C			PSYC
Pinaceae	*Picea abies* (L.) H.Karst.	Fichtenrinde		W			DIGE
	*Pinus sylvestris* L.	*Pinus sylvestris*	Manna pu	W, (C) ^F^	DIGE, ENDO, SKIN		
Piperaceae	*Piper nigrum* L.	*Piper nigrum*^L^, Pfeffer ^AL^	Walge ja must pippar	P	DIGE		DIGE, EAR, RESP, SKIN
Plantaginaceae	*Linaria vulgaris* Mill.	*Linaria vulgaris*		W ^F^			SKIN
	*Plantago major* L.	*Plantago major*	Tee lehhed	W ^F^	SKIN		DIGE, SKIN, UROL
	*Veronica agrestis* L.	*Veronica agrestis*		W			PSYC
	*Veronica arvensis* L.	*Veronica arvensis*		W			PSYC
	*Veronica beccabunga* L.	*Veronica beccabunga*		W ^F^			GEUN, MUSC
	*Veronica longifolia* L.	*Veronica longifolia*		W			SKIN
	*Veronica officinalis* L.	*Veronica officinalis*	Jooksja rohhi, jaani rohhi, mailase rohhi	W ^F^	CULT, ENDO, GEUN, SKIN		
Poaceae	*Alopecurus pratensis* L.	Roggengras		W, C ^F^			GEUN
	*Avena sativa* L.	Haferkörner, Haferstroh		(W, C) ^F^			DIGE, RESP
	*Briza media* L.	*Briza media*		W			DIGE, GEUN
	*Calamagrostis* sp.	*Calamagrostis*		W			GENI
	*Hordeum vulgare* L.	Gerstenkörner, Gerstengrütze		C			EYE, SKIN
	*Secale cereale* L.	Roggenblüthe, Roggenähren, Roggenmehl, Roggenbrod		C			DIGE, RESP, SKIN
	*Triticum* sp.	Weizenmehl		(W, (C)) ^F^			SKIN
Polygalaceae	*Persicaria maculosa* Gray	*Polygonum persicaria*		W ^F^			SKIN
	*Polygala amara* L.	*Polygala amara*		W ^F^			PSYC
	*Polygala* sp.	*Polygala*		(W, C) ^F^			GEUN, PSYC
	*Polygala vulgaris* L.	*Polygala vulgaris*		W ^F^			GENI
Polygonaceae	*Rumex crispus* L.	*Rumex crispus*		(W) ^F^			DIGE, SKIN
	*Rumex obtusifolius* L.	*Rumex obtusifolius*	Hobbosehoblikad	W	DIGE, SKIN		
Polypodiaceae	*Dryopteris filixmas* (L.) Schott	*Aspidium filix mas*		W			SKIN
	*Polypodium vulgare* L.	*Equisetum fragile*, Engelsüß	Rinna rohhi	W ^F^	RESP		
Primulaceae	*Lysimachia vulgaris* L.	*Lysimachia vulgaris*		W, (C ^F^)			DIGE
Ranunculaceae	*Aconitum napellus* L.	*Aconitum napellus*		W			SKIN
	*Actaea spicata* L.	*Actaea spicata*	Akkitse haiguse rohi	W ^F^	GEUN, PSYC		PSYC
	*Anemone nemorosa* L.	*Anemone nemorosa*	Külma ellased	W ^F^	EYE, SKIN		
	*Caltha palustris* L.	*Caltha palustris*	Warsa kabjad, kuller kuppud	W ^F^	DIGE		
	*Consolida regalis* Gray	*Delphinium consolida*		W, (C ^F^)			DIGE, RESP
	*Ranunculus ficaria* L.	*Ranunculus ficaria*		W, (C) ^F^			CARD
	*Ranunculus acris* L.	*Ranunculus acris*	Tullikad, sobia rohhi, jooksja rohhi, pöld ingwerid, tullililled	W ^F^	CARD, ENDO, GEUN, MUSC, SKIN		SKIN
Rhamnaceae	*Frangula alnus* Mill.	*Rhamnus frangula*		W, (C) ^F^			DIGE, MUSC, SKIN, UROL
	*Rhamnus cathartica* L.	*Rhamnus cathartica*	Paaks pu	W, (C) ^F^	DIGE, SKIN		RESP
Rosaceae	*Argentina anserina* (L.) Rydb.	*Hippocrepis comosa*	Hoolmete rohhi	W	DIGE		
	*Comarum palustre* L.	*Comarum palustre*		W ^F^			MUSC
	*Filipendula ulmaria* (L.) Maxim.	*Filipendula ulmaria*^L^, *Spiraea ulmaria*^AL^	Wormid, naeste rohhi	W ^F^	PCFP		DIGE, EYE, NEUR, SKIN
	*Filipendula vulgaris* Moench	*Spiraea vulgaris*		W, C^F^			DIGE
	*Fragaria vesca* L.	*Fragaria vesca*		W, (C) ^F^			DIGE, RESP
	*Geum urbanum* L.	*Geum urbanum*		W ^F^			GENI
	*Malus* sp.	Apfelbaumblätter, Sauere Aepfel		W/C			GEUN, SKIN
	*Malus sylvestris* (L.) Mill.	wilder Apfelbaum		W, C ^F^			SKIN
	*Potentilla erecta* (L.) Raeusch.	*Tormentilla*		W ^F^			DIGE, MUSC
	*Prunus cerasus* L.	*Prunus cerasus*		W, C ^F^		PCFP	PCFP
	*Prunus domestica* L.	Pflaumensaft		C			PCFP
	*Prunus padus* L.	*Prunus padus*		W, C ^F^		GEUN, SKIN	CARD, NEUR, UROL
	*Rubus caesius* L.	*Rubus caesius*		W ^F^			CARD
	*Rubus chamaemorus* L.	*Rubus chamaemorus*	Murrakad, (kabbarad, kaas marjad)	W ^F^	CARD		
	*Rubus idaeus* L.	*Rubus idaeus*		W, C ^F^			RESP, SKIN
	*Rubus saxatilis* L.	*Rubus saxatilis*		W ^F^			MUSC
	*Sorbus aucuparia* L.	*Sorbus aucuparia*		W, C ^F^			GEUN, MUSC
Rubiaceae	*Galium odoratum* (L.) Scop.	*Asperula*		(W) ^F^			DIGE
	*Galium boreale* L.	*Galium boreale*	Maddarad	W ^F^	GENI		
Sapindaceae	*Aesculus hippocastanum* L.	*Aesculus hippocastanum*		C ^F^			MUSC
Saxifragaceae	*Chrysosplenium alternifolium* L.	*Chrysosplenium alternifolium*		W			DIGE, SKIN
Scrophulariaceae	*Scrophularia nodosa* L.	*Scrophularia*		W			CARD
	*Verbascum thapsus* L.	*Verbascum thapsus*	Ühheksa mehhe wäggi	W ^F^	RESP, SKIN	RESP	GEUN, MUSC, SKIN
Solanaceae	*Capsicum annuum* L.	*Capsicum annuum*	Türgi pippar	P	GEUN		DIGE, GEUN, MUSC
	*Datura stramonium* L.	*Datura stramonium*		C, (W) ^F^			NEUR, RESP
	*Hyoscyamus niger* L.	*Hyoscyamus niger*	Hüllo koera rohhi, hüllo koera hänna rohhi	W ^F^	DIGE		DIGE, MUSC, NEUR, PSYC, SKIN
	*Nicotiana rustica* L.	*Nicotiana rustica*^L AL^, *Nicotiana tabac. Rustica*^AR^	Tubbaka lehhed	C	DIGE, SKIN	EYE, SKIN	DIGE, GEUN, RESP, SKIN
	*Solanum americanum* Mill.	*Solanum nigrum*		W ^F^			PSYC
	*Solanum dulcamara* L.	*Solanum dulcamara*	Solika rohhi	W, (C) ^F^	DIGE		DIGE, GEUN
	*Solanum tuberosum* L.	*Solanum tuberosum*		C			GEUN, NEUR, SKIN
Taxaceae	*Taxus baccata* L.	*Taxus baccata*	Juhha pu	W, (C) ^F^	SKIN		
Thymelaeaceae	*Daphne mezereum* L.	*Daphne mezereum*		W, C ^F^			DIGE, GEUN
Urticaceae	*Urtica urens* L.	*Urtica urens*		W ^F^			MUSC, NEUR, RESP, SKIN, UROL
Viburnaceae	*Sambucus ebulus* L.	*Sambucus ebulus^,^*		W, C ^F^			SKIN
	*Sambucus nigra* L.	*Sambucus niger*		W, C ^F^			CARD, SKIN
	*Viburnum opulus* L.	*Viburnum opulus*		W, C ^F^			SKIN
Violaceae	*Viola tricolor* L.	*Viola tricolor*	Mailase rohhi	W	SKIN		RESP
	*Viola arvensis* Murray	*Viola arvensis*		W			PSYC
Zingiberaceae	*Aframomum melegueta* K.Schum.	*Grana paradisi*		P			DIGE
	*Alpinia galanga* (L.) Willd.	*Alpinia galanga*	Jalgendi jured	P	PCFP		
	*Alpinia officinarum* Hance	*Radix Galangae*		P			DIGE
	*Curcuma zedoaria* (Christm.) Roscoe	*Curcuma zedoaria*		P			DIGE
Zygophyllaceae	*Guaiacum officinale* L.	*Tinct. Guajaci*	Plussas drape	P			NEUR
Unidentified	Unidentified	Speedeja sahle	speedeja sahle				GEUN

Name in original if listed by more than one source: (L) Luce, (AL) Alksnis, and (AR) Aronson. Origin of plant: if different in Friebe [40] (F): wild (W), cultivated (C), or purchased (P). Abbreviations of disease categories: BLIM—Blood, Blood Forming Organs and Immune Mechanism, CARD—Cardiovascular, CULT—Culture Bound Syndrome, DIGE—Digestive, EAR—Ear, ENDO—Endocrine/Metabolic and Nutritional, EYE—Eye, GENI—Female Genital, GEUN—General and Unspecified diseases, MUSC—Musculoskeletal, NEUR—Neurological, PCFP—Pregnancy, Childbearing, Family Planning, PSYC—Psychological, RESP—Respiratory, SKIN—Skin.

**Table 2:** Comparison of the three authors.

	Luce 1829	Aronson 1891	Alksnis 1894
Taxa	66	19	186
DUR	123	35	445
UI	99	31	325
Most-diversely used species (DUR)	*Ranunculus acris* (9), *Hypericum perforatum* (7), *Artemisia absinthium* (5)	*Valeriana officinalis* (6), *Cinnamomum camphora* (4), *Matricaria chamomilla* (3)	*Allium cepa* (10), *Urtica urens* (10), *Betula* spp. (9)
Most-mentioned etic disease categories (DUR)	SKIN (38), DIGE (33), GEUN (10), ENDO (9)	GEUN (9), DIGE (9), SKIN (5), RESP (2)	DIGE (104), SKIN (94), RESP (64), GEUN (46)

**Table 3:** Jaccard Indexes (JI) showing the proportional overlap between the authors.

Taxa/UI	Luce 1829	Aronson 1891	Alksnis 1894
Luce 1829	X	0.07438	0.06
Aronson 1891	0.214286	X	0.040936
Alksnis 1894	0.193878	0.084656	X

## Data Availability

https://doi.org/10.5281/zenodo.6106746 (accessed on 2 February 2022).
